# Using health service data to understand unregulated toxic drug deaths in Alberta (2018–2023): an ecological study

**DOI:** 10.24095/hpcdp.46.7/8.02

**Published:** 2026-07

**Authors:** Holly Mathias, Karen V. Lithgow, Euan Thomson, Claire Benny, Esther Tailfeathers, Elaine Hyshka

**Affiliations:** 1 Department of Human Ecology, University of Alberta, Edmonton, Alberta, Canada; 2 Inner City Health and Wellness Program, University of Alberta, Edmonton, Alberta, Canada; 3 Department of Microbiology & Immunology, Dalhousie University, Halifax, Nova Scotia, Canada; 4 Independent Researcher, Calgary, Alberta, Canada; 5 School of Public Health, University of Alberta, Edmonton, Alberta, Canada; 6 Kainai First Nation, Alberta, Canada

**Keywords:** drug overdoses, emergency medical services, harm reduction, substance-related harms, opioids

## Abstract

**Introduction:**

Ten years into the most acute phase of the toxic drug crisis in Canada, trends in drug-related deaths remain poorly understood. This is hindered by delayed reporting of drug deaths in some provinces, including Alberta. We used publicly available emergency medical services (EMS) and supervised consumption site (SCS) utilization data to examine their associations with drug poisoning deaths to assess if these measures could serve as reliable proxies for fatalities to support evidence-based public health responses.

**Methods:**

Data on Alberta-wide EMS dispatches for opioid poisoning, Alberta-wide drug deaths, and SCS drug poisoning events at the SCS in Calgary from January 2018 to March 2023 were analyzed. We used linear regression modelling to investigate the association between EMS dispatches and drug deaths (by month) and between SCS drug poisoning events and drug deaths (by month).

**Results:**

Significant associations were found between drug-related deaths in Alberta and both the number of EMS dispatches (β = .06, 95% CI: 0.05–0.06) and SCS drug poisoning events (β = .73, 95% CI: 0.57–0.88), suggesting these measures mirror drug-related deaths in the province. Inverse associations were also found between drug-related deaths and SCS visits as well as visits in which drugs were consumed.

**Conclusion:**

Drug poisoning events recorded in EMS or SCS data may serve as a proxy for understanding trends in drug deaths. These alternative data are often publicly available and may help inform public health response to periods of increased death risk when official death data are delayed or unavailable.

## Highlights

• Publicly available data on unregulated toxic drug poisoning deaths in Alberta are often delayed or incomplete.

• Emergency medical service dispatch data and supervised consumption site drug-related event data are potential proxies for drug deaths on a provincial level.

• Using other health service data can provide insight into drug poisoning death trends and support public health personnel and response planning.

## Introduction

The toxic drug crisis is an urgent Canadian public health issue, with increasing mortality and morbidity associated with the use of an unregulated drug supply contaminated with clandestinely produced fentanyl and other substances.^1^^,^^2^ Since January 2016, more than 53 000 Canadians have died from drug poisoning, including more than 10 500 Albertans.^2^ Alberta, British Columbia and Ontario account for 87% of all drug-related deaths in Canada.^2^ Notably, due to colonial violence, discrimination and racism, First Nations people disproportionately account for drug-related deaths in Alberta, prompting multiple state-of-emergency declarations in some communities.^3^^-^^6^

The Government of Alberta publicly shares drug death data. Preliminary fatality counts typically lag by three to four months, while EMS dispatches are usually released with less than a month’s lag. However, public EMS opioid-related dispatch reports are often incomplete as they lack non-opioid drug poisoning events and other emergency service dispatches (e.g. fire and police departments). They also include all dispatches to an opioid-related event which may or may not result in transport to hospital.^7^ Meanwhile, questions have been raised about the accuracy of fatality numbers.^3^^,^^8^^-^^11^ The Public Health Agency of Canada reports an average delay of six months between the occurrence of a death and its inclusion in national death data.^12^ In Alberta, this delay can reach nine months, and since 2021, Alberta has been the only province or territory not reporting stimulant deaths to the federal government.^2^^,^^13^

Without timely, accessible and high-resolution data, it is difficult to implement interventions that effectively respond to this public health emergency. Although there was a year-over-year decrease in total drug-related deaths from 2024 to 2025, this decrease has not been experienced uniformly.^2^ Since 2025, there has been a sharp increase in carfentanil-related deaths, and some cities, such as Edmonton, continue to experience record drug-related deaths.^14^ Given this ongoing public health crisis and the lack of available and transparent data, this study aimed to determine whether additional health service data (i.e. EMS dispatches for drug poisonings and drug poisonings that occur during SCS utilization) can be used to understand trends in drug deaths in Alberta, by serving as a proxy for unregulated drug supply toxicity. Supplementing fatality trends with data from these health services could help guide targeted responses that prevent further deaths.

While EMS data are collected and reported publicly with less lag time, public transparency on SCS data has been significantly curtailed since 2024.^15^ Both services engage a large number of people who use drugs daily.^16^ EMS are dispatched to hundreds of drug poisoning events weekly in Alberta.^3^^,^^17^ SCSs are evidence-based harm reduction interventions that provide a hygienic place to consume pre-obtained drugs, and access to wraparound health and social services, such as primary care and housing supports.^18^ As of February 2026, there are 32 SCSs in Canada, supported by global evidence on their effectiveness of preventing drug poisoning deaths and supporting public safety.^19^^,^^20^ However, no research has considered the utility of SCS and EMS data as proxies for drug death trends. A proof-of-concept study used Ottawa Paramedic Service data to provide epidemiologic data on municipal opioid overdoses and demonstrated that EMS data were beneficial for assessing trends in the unregulated drug supply but called for further validation.^21^ An ecological study and spatial analysis using data from Toronto, Ontario neighbourhoods demonstrated the association between municipal SCS implementation and reduced deaths.^22^ This study provides further validation using health services data (from EMS and SCS) to understand current drug deaths trends. Specifically, we aim to understand:

1) what are the statistical correlations between adverse events and utilization at SCSs in Alberta and Calgary, and the respective rates of drug poisoning deaths in the province and city?

2) is there a statistically significant correlation between the number of EMS dispatches for suspected drug poisonings in Alberta and the overall number of drug poisoning deaths in the province?

## Methods

### Study design and time period

This secondary data analysis investigates drug deaths in Alberta from 1 January 2018 to 31 December 2023 and the link to SCS utilization, SCS drug poisoning events, and opioid-related EMS dispatches.

### Study setting

Alberta is a province in Western Canada (population: 5 million) that has experienced some of the highest annual drug poisoning deaths in Canada (2140 deaths in 2023).^2^^,^^23^ Despite this, Alberta is shifting away from a multipronged drug poisoning prevention strategy that includes evidence-based harm reduction interventions such as SCSs. The new approach, termed the “Alberta Recovery Model,” prioritizes abstinence-based and recovery-oriented approaches to care, including a highly criticized plan to enact compulsory bed-based treatment and forced abstinence through the *Compassionate Intervention Act.* As a result of these changes in political ideology, harm-reduction tactics have been de-emphasized and several SCSs have been closed in tandem.^24^^-^^27^ The high rates of drug poisoning have also created considerable pressure on the strained EMS system.^28^ In 2024, overtime among Albertan paramedics increased 81% compared to 2021, and there was an average of 40 unfilled shifts per day in Edmonton.^29^^,^^30^

Ethics approval was provided by University of Alberta Health Research Ethics Board – Health Panel (Pro00133344).

### Data sources

Secondary data were collected in aggregate from the Government of Alberta Provincial Substance Use Surveillance Dashboard, the Alberta Health Services SCS Monthly Reports for Calgary SCS, and Alberta Health Services – EMS Research and Science (see Table 1 for data sources and variables).

**Table 1:** Additional information on data sources

**Data source and period**	**Variable of interest**	**Variable notes**
Government of Alberta Provincial Substance Use Surveillance Dashboard (PSUSD); January 2018–December 2023	Drug poisoning deaths in Alberta	“Any substance”, Alberta-wide monthly counts
	EMS responses to opioid-related events	Monthly counts, Alberta-wide
	Rate of opioid responses to total EMS responses (%)	Calculated from EMS responses to opioid-related events (PSUSD) and total emergency events (PSUSD)
	Drug poisoning rate	“Any substance”, Alberta-wide monthly rates per 100 000 population
	Drug poisoning deaths_Calgary	Monthly counts, Calgary only
	Drug poisoning rate_Calgary	“Any substance”, monthly rates per 100 000 population for Calgary municipality
	Drug poisoning deaths Alberta not Calgary	Calculated from Alberta and Calgary monthly counts (subtracted Calgary counts from Alberta counts)
Alberta Health Services monthly reports for Calgary SCS; January 2018–December 2023	Client_visits	The number of clients who visit per month, as recorded and reported by Calgary SCS staff
	Drug_consumption_events	Monthly drug consumption events, Calgary SCS
	Overdoses	Monthly overdoses, Calgary SCS
	Poisoning_rate_%	Drug poisoning rate (overdoses per number of times drugs consumed), Calgary SCS
Alberta Health Services – EMS Research and Science; January 2018–March 2023	Total EMS emergency events	Monthly counts; Alberta-wide
	Overdose/Poisoning emergency events	For total emergency events, Overdose/Poisoning events were identified by Card Type 23; Alberta-wide
	Overdose as a proportion of all events	Monthly counts, Overdose/Poisoning as a proportion of total EMS events; Alberta-wide

**Abbreviations:** EMS, emergency medical services; PSUSD, provincial substance use surveillance dashboard; SCS, supervised consumption site.

Under federal law, SCSs are distinguished from overdose prevention sites (OPSs) by authorization pathway and typically are more permanent and offer a larger number of ancillary services compared to OPSs. In this study, SCS utilization refers to the number of drug consumption events within the site. SCS drug poisoning events refer to drug consumption events that necessitate a medical response, including naloxone or oxygen administration. It should be noted that none of these events in Alberta have been fatal. EMS dispatches refer to events where EMS are dispatched to respond to a potential opioid-related event.

Drug poisoning events reported at SCSs are expected to function as a proxy for the toxicity of the unregulated (illegal) drug supply, given that SCS clients bring unregulated drugs into SCSs for consumption. Quarterly data on drug poisoning events and rate of drug poisoning were only available for the full study period (2018–2023) for Calgary and Edmonton. Given that the Calgary SCS comprises the highest proportion of province-wide SCS visits and most complete data available during the study period, the publicly available Calgary SCS data were used to investigate associations between drug poisonings in SCSs and drug death rates in the municipality of Calgary. Data were limited to shorter time frames for other sites including Grand Prairie (2019 Q1–2023 Q4), Lethbridge AHS OPS (2020 Q3–2023 Q4), Lethbridge ARCHES (2018 Q1–2020 Q3) and Red Deer (2018 Q4–2023 Q4). SCS utilization across Alberta was calculated by summing quarterly counts from all sites for each variable (see Table 2). From the site’s opening in 2017, Calgary SCS was the only site publicly posting monthly reports that included the number of consumption events and drug poisonings during consumption, but these were removed from public access in October 2024 and further reporting was halted.^15^ However, visits to the Calgary SCS represented greater than one-fifth of total provincial SCS visits for the full study period, and drug deaths in Calgary made up 23% to 43% of the provincial total. Focussing on the detailed Calgary data provides a wide-angle view of the provincial crisis from the province’s largest city.

**Table 2:** Comparison of drug deaths and related events in Calgary and Alberta

**Year**	**Calgary**		**Alberta**	
	**Monthly drug deaths^a^**			
	Deaths (SD)	Deaths per 100 000 (SD)	Deaths (SD)	Deaths per 100 000 (SD)
2018–2023	41.5 (14.7)	34.4 (10.9)	123.4 (44.5)	33.0 (11.3)
2018	27.8 (6.6)	28.4 (4)	79.7 (9.1)	22.3 (2.6)
2019	30.2 (12.8)	21.4 (6.2)	66.7 (13.1)	18.4 (3.6)
2020	43.5 (4.8)	32.9 (9.5)	117.3 (33.4)	31.9 (9.1)
2021	49.5 (11.2)	41.4 (7.3)	157.2 (22.1)	42.6 (6)
2022	56.6 (12.1)	38.2 (8.7)	146.9 (20.9)	39.1 (5.6)
2023	41.3 (14.5)	44.3 (9.8)	172.4 (18.7)	44.1 (4.8)
**Year**	**Quarterly supervised consumption sites usage^b^**			
	Drug poisoning events	Drug poisoning rate^c^	Drug poisoning events	Drug poisoning rate^c^
2018–2023	340 (164.6)	3.2 (2.2)	1052.3 (371.3)	2.2 (1.1)
2018	179 (35.6)	1.5 (0.3)	619 (396.9)	1.2 (0.2)
2019	181.8 (16.5)	1.1 (0.1)	1034.3 (36.7)	1 (0.1)
2020	227.5 (81.9)	1.8 (0.9)	806 (298.1)	1.6 (0.7)
2021	518.5 (97.9)	5.0 (1.5)	1423.8 (194.4)	3.2 (0.4)
2022	404.3 (93)	4.1 (1.1)	1174.3 (371.5)	2.6 (0.8)
2023	529 (25.9)	5.9 (0.4)	1256.8 (205.6)	3.4 (0.4)
**Year**	**Monthly emergency medical services (EMS) usage^a,d^**			
	Dispatches for opioid poisoning	% of EMS dispatches for opioid poisoning	Dispatches for opioid poisoning	% of EMS dispatches for opioid poisoning^e^
2018–2023^f^	N/A	N/A	1854.5 (668.5)	5.4% (1.5)
2018	N/A	N/A	1303.1 (123.1)	4.4% (0.4)
2019	N/A	N/A	1190.4 (130.5)	3.9% (0.4)
2020	N/A	N/A	1601.3 (326.1)	5.0% (0.9)
2021	N/A	N/A	2403.5 (540.4)	6.6% (1.0)
2022	N/A	N/A	2492.8 (229.3)	6.7% (0.7)
2023	N/A	N/A	N/A	N/A

**Source:** 2018–2023 Alberta Health Services SCS monthly reports for Calgary SCS and Government of Alberta Provincial Substance Use Surveillance Dashboard.

**Abbreviations:** EMS, emergency medical services; SCS, supervised consumption site.

**Note:** Data expressed as monthly or quarterly means with standard deviation shown in brackets, (SD).

^a^ Means calculated from monthly data aggregated across the time frame.

^b^ Means calculated from quarterly data aggregated across the time frame, provincial data were aggregated from all available data from supervised consumption sites across the province.

^c^ Rate of drug poisoning events as a proportion of drug consumption events.

^d^ Disaggregated EMS usage data not available for Calgary; only aggregated EMS at the provincial level were accessible.

^e^ Dispatches for opioid poisoning expressed as a percentage of total EMS dispatches.

^f^ Data only available from January 2018 to March 2023.

## Measures

### Primary outcome

The primary outcome is provincial drug poisoning by any substance per month.

### Exposures

Exposure variables are the Alberta-wide monthly counts of total EMS emergency events, poisoning emergency events, and poisoning emergency events as a proportion of all EMS events. Additionally, Calgary SCS data on monthly client visits, drug consumption events, poisonings, and drug poisoning rates (poisonings per number of times drugs consumed) were analyzed.

### Covariate

We controlled for seasonality in months based on literature that highlights seasonality in drug deaths.^31^

## Data analysis

Drug poisoning rates at the Calgary SCS were calculated by normalizing drug poisoning events to total drug consumption events for the same time frame. Associations between variables were assessed with a generalized linear regression model and Spearman’s correlation. We used multiple linear regression modelling to examine the association between Calgary SCS utilization (site visits, drug consumption events, poisoning events, poisoning rate) and drug poisoning deaths in Alberta and the association between drug EMS dispatches (total, opioid-related, and non-opioid-related) and poisoning deaths in Alberta. All regression models were adjusted for seasonality. Statistics were performed in GraphPad Prism (GraphPad, San Diego, CA) and Stata18 (StataCorp, College Station, TX). Graphs were prepared in GraphPad Prism v10.

## Results

From 1 January 2018 to 31 December 2023, a monthly average of 123.4 drug deaths (33.0 deaths per 100 000 people) was observed in Alberta, while a monthly average of 41.5 drug deaths (34.4 deaths per 100 000 people) was observed in Calgary (Table 2). Provincially, drug deaths peaked in 2023 with a monthly average of 172.4 deaths (44.1 deaths per 100 000 people). In Calgary, the monthly average for drug deaths was 41.3, at a rate of 44.3 deaths per 100 000 people. Drug deaths in Calgary represented 23% to 43% of the total in Alberta throughout the study period.

### Association between drug poisoning deaths and supervised consumption site events in Alberta

Publicly available data from each SCS in Alberta revealed a quarterly provincial average of 1052 drug poisoning events (2.2 drug poisoning events per consumption event) from 2018 to 2023 (Table 1, Figure 1). The association between quarterly provincial drug poisonings during supervised consumption and drug poisoning deaths (per 100 000) showed close alignment (Figure 1C) and strong positive correlation from 2018 to 2023 (**ρ** = 0.94, *p* < 0.0001; Figure 1D). Quarterly drug poisoning events reported across all Alberta SCS/OPS exhibited a moderate positive correlation with quarterly provincial drug deaths (**ρ** = 0.59, *p* = 0.0024; Figure 1E). Notably, total site visits to Alberta SCS were negatively correlated with provincial drug deaths (**ρ** = −0.59, *p* = 0.0024; Figure 1F).

**Figure 1:**
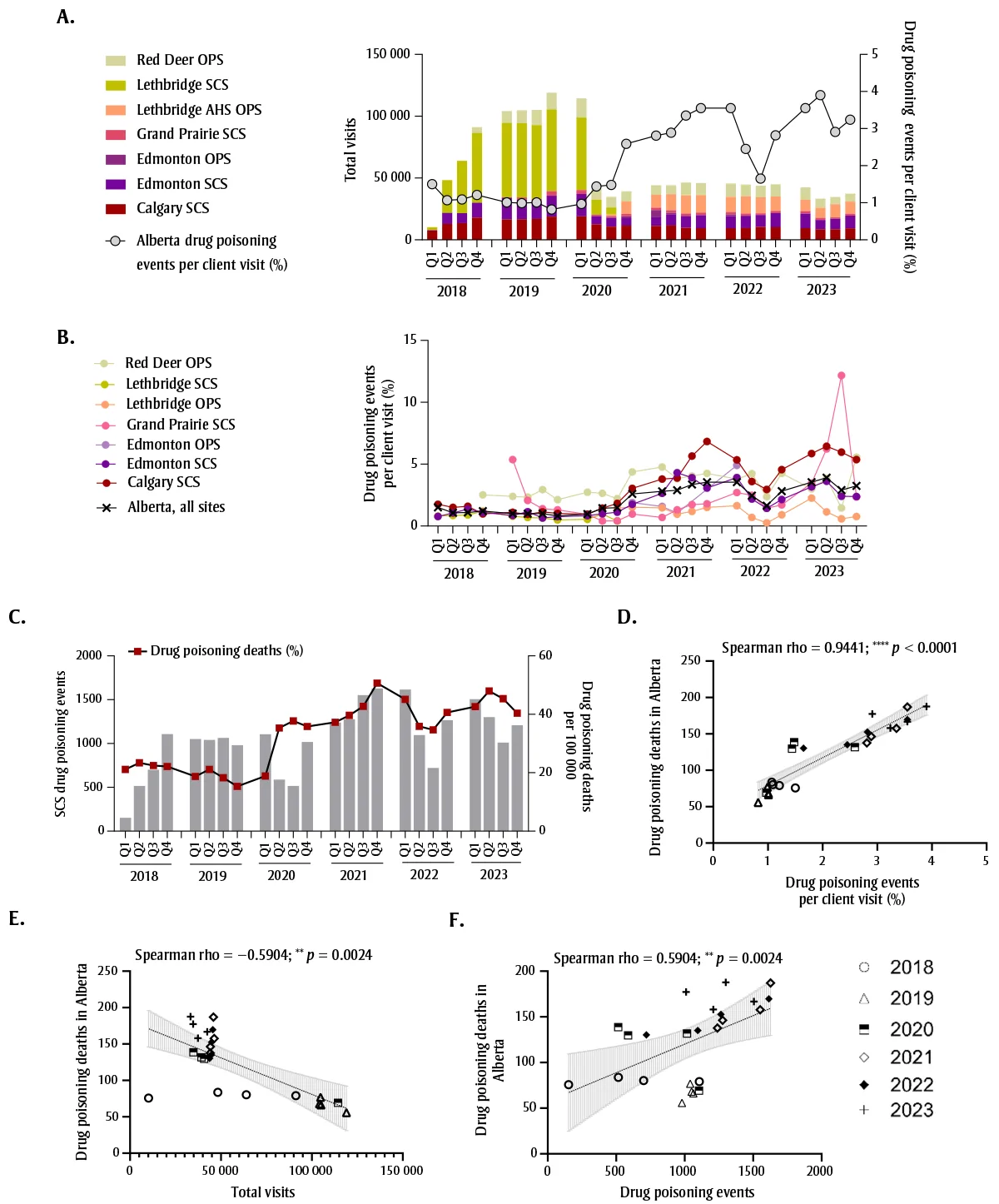
Associations between drug poisoning events and supervised consumption site visits and events, Alberta, Canada **Abbreviations:** AHS, Alberta Health Services; OPS, overdose prevention site; SCS, supervised consumption site. Notes: **(A)** Quarterly number of visits (bars) per supervised consumption site across Alberta from January 2018 to December 2023, colour coded by site, overlaid with the total province-wide drug poisoning events (%) (circles; right y-axis) expressed as drug poisonings recorded as a percentage of total client visits at supervised consumption sites across Alberta. **(B)** Quarterly drug poisoning events (%) at supervised consumption sites across Alberta from 2018 to December 2023, colour coded by site, overlaid with the Alberta drug poisoning events (%) (X). **(C)** Quarterly number of drug poisoning events recorded at all supervised consumption sites across Alberta (bars), overlaid with the drug poisoning deaths (%) across Alberta per 100 000 people from 2018 to December 2023 (red squares; right y-axis). **(D–F)** Scatterplots of drug deaths in Alberta as a function of the following variables at supervised consumption services: **(D)** drug poisoning events per client visit (%), and **(E)** total client visits, and **(F)** total drug poisoning events. Individual data points correspond to quarterly data collected across all provincial SCSs from January 2018 to December 2023, with symbols denoting the year. Regression lines were fitted using a generalized linear model and error bars indicate 95% confidence intervals. Associations were assessed by Spearman’s correlation, with the rho (ρ) value and *p* value shown above each scatterplot.

## Association between drug poisoning deaths and supervised consumption site utilization in Calgary, Alberta

At the onset of the COVID-19 pandemic, the proportion of drug consumption events at Calgary SCS that resulted in a drug poisoning event increased (Figure 2A). Although visits to Calgary SCS started to decline in the first months of the pandemic through 2023, there was an increase in SCS drug poisoning events from April to December 2021. Drug poisoning at Calgary SCS moved in tandem with death patterns in Calgary. There was a negative correlation between monthly municipal drug deaths and both site visits (**ρ** = −0.67, *p* < 0.0001; Figure 2B) and drug consumption events (**ρ** = −0.66, *p* < 0.0001; Figure 2C) at Calgary SCS. Monthly drug poisoning deaths in Calgary also correlated with the number of drug poisoning events reported at Calgary SCS (**ρ** = 0.70, *p* < 0.0001; Figure 2D) and the proportion of drug consumption events with a drug poisoning at Calgary SCS (**ρ** = 0.78, *p* < 0.0001; Figure 2E).

**Figure 2:**
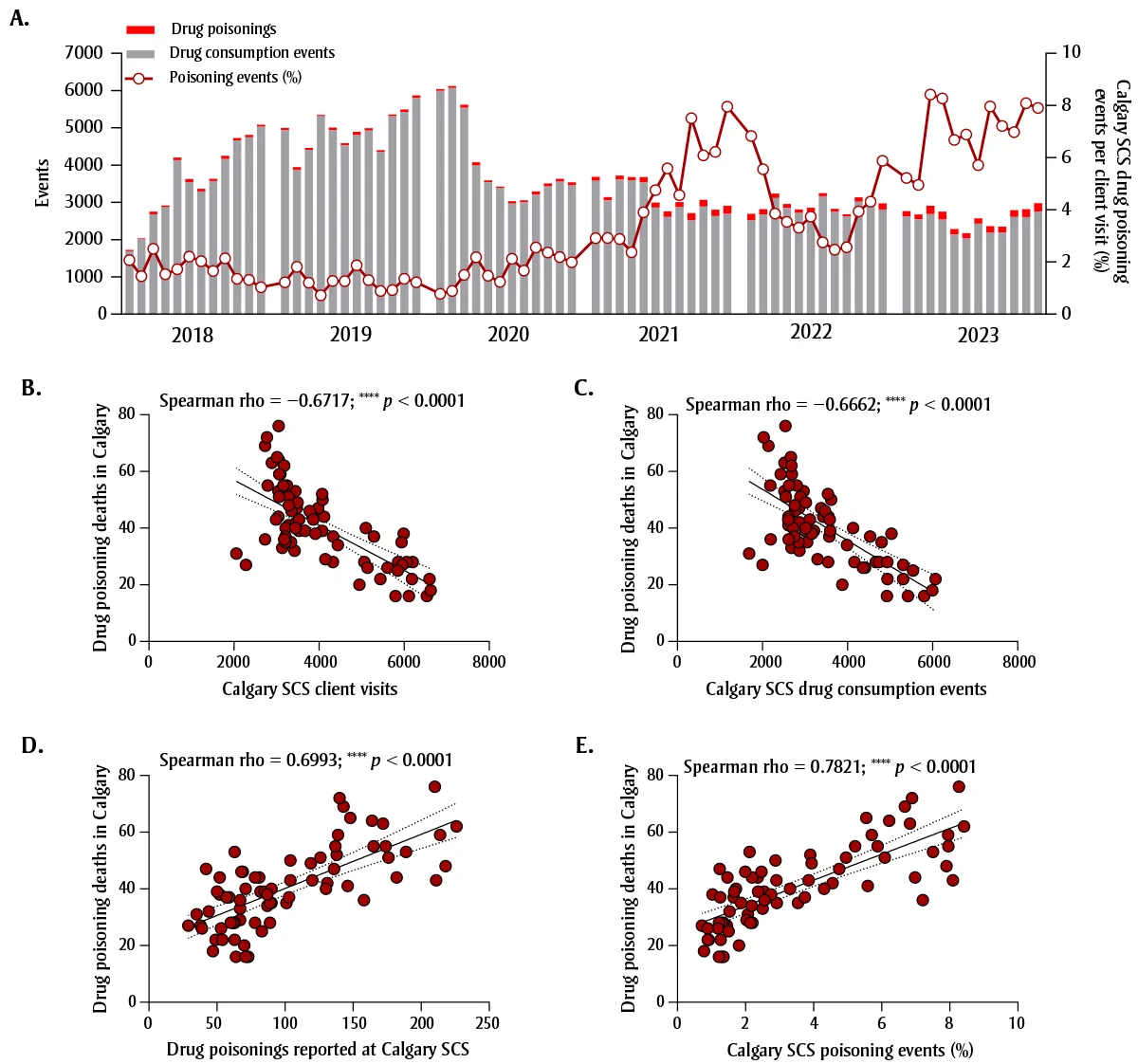
Associations between drug poisoning deaths and supervised consumption site visits in Calgary, Alberta, Canada **Abbreviation:** SCS, supervised consumption site. Notes: **(A)** Monthly number of drug consumption events (grey) and drug poisoning events (red) recorded at Calgary SCS from January 2018 to December 2023, overlaid with the Calgary SCS poisoning events expressed as the percentage of drug poisoning events relative to drug consumption events (red circles; right y-axis). **(B–E)** Scatterplots of drug poisoning deaths in Calgary as a function of Calgary SCS **(B)** client visits, **(C)** drug consumption events, **(D)** drug poisoning events and **(E)** drug poisoning events (%). Individual data points correspond to monthly data collected from January 2018 to December 2023. Regression lines were fitted using a generalized linear model and error bars indicate 95% confidence intervals. Associations were assessed with Spearman’s correlation, with the rho (ρ) value and *p* value indicated above each scatterplot.

## Association between drug poisoning deaths in Alberta and Calgary SCS utilization

The municipality of Calgary and the province of Alberta showed similar monthly patterns of drug deaths and drug death rates per 100 000 from 2018 to 2023 (Figure 3A). The frequency of drug poisoning events under supervised consumption at Calgary SCS strongly correlated with total monthly drug deaths in Alberta (**ρ** = 0.88, *p* < 0.0001; Figure 3B), even when data from the Calgary municipality were removed (**ρ** = 0.86, *p* < 0.0001; Figure 3C).

**Figure 3:**
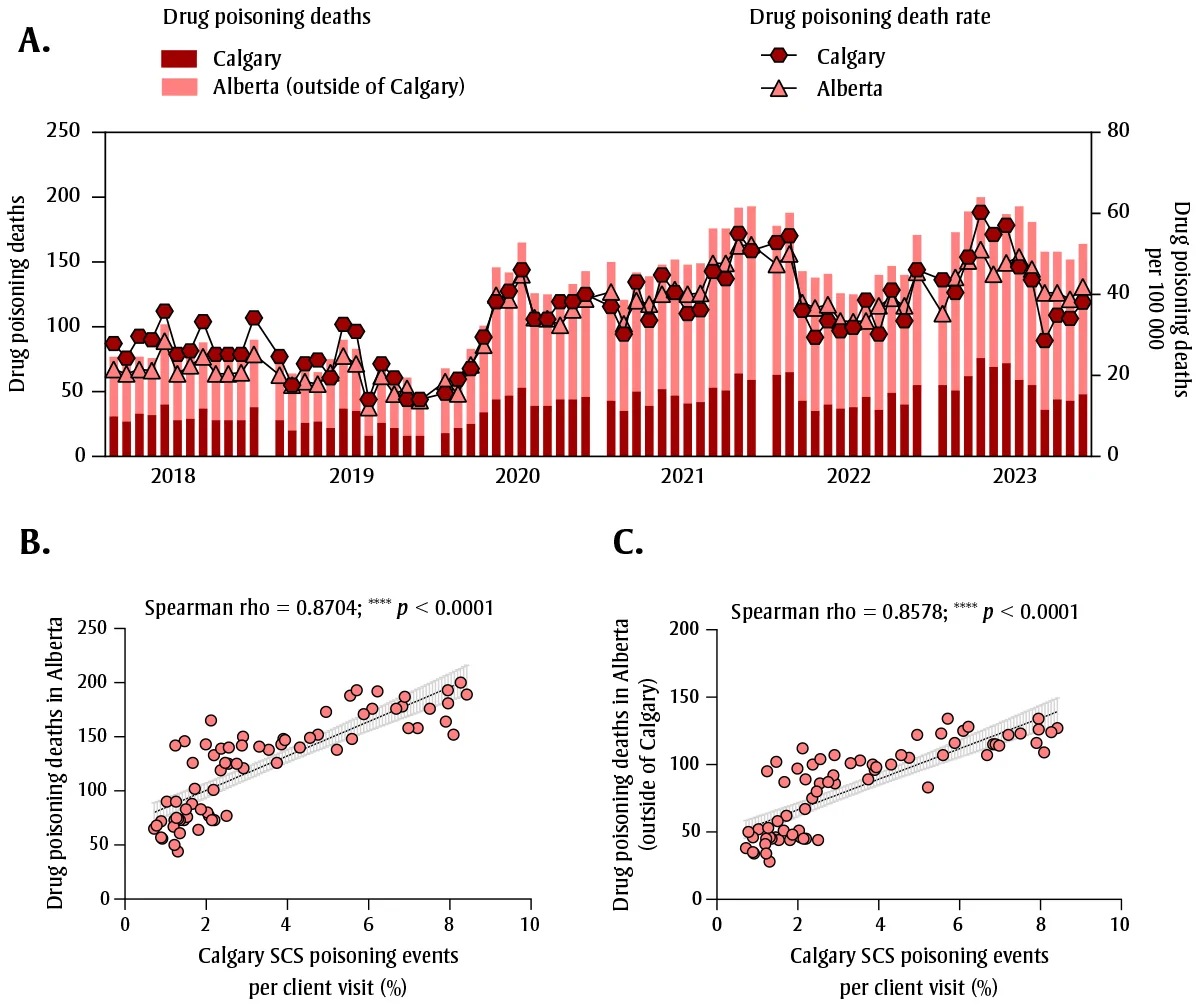
Associations between province-wide drug poisoning deaths in Alberta and supervised consumption site visits in Calgary, Alberta, Canada **Abbreviation:** SCS, supervised consumption site. Notes: **(A)** Monthly drug poisoning events in Calgary (red) and Alberta, not including Calgary (pink) from January 2018 to December 2023, overlaid with the drug death rate in Calgary (red circle; right y-axis) or Alberta (pink triangle, right y-axis). **(B)** Scatterplot of drug deaths in Alberta as a function of Calgary SCS poisoning events per client visit (%). **(C)** Scatterplot of drug deaths in Alberta (Calgary excluded) as a function of Calgary SCS poisoning events per client visit (%).

Individual data points correspond to monthly data collected from January 2018 to December 2023. Regression lines were fitted using a generalized linear model and error bars indicate 95% confidence intervals. Associations were assessed with Spearman’s correlation, with the rho (ρ) value and *p* value indicated above each scatterplot.

## Association between drug poisoning deaths and EMS dispatches in Alberta

Across Alberta during the study period, there was a monthly average of 1854 EMS dispatches for drug poisoning events at a rate of 5.4% of the total EMS dispatches (Table 1). EMS dispatches for drug poisoning events peaked in 2022 with a monthly average of 2493 dispatches for drug poisoning events (6.7% of total EMS dispatches, Table 1). Provincial drug poisoning deaths across the study period positively correlated with total EMS dispatches (**ρ** = 0.75, *p* < 0.0001; Figure 4C) and EMS dispatches for opioid-related events (**ρ** = 0.91, *p* < 0.0001; Figure 4D).

**Figure 4:**
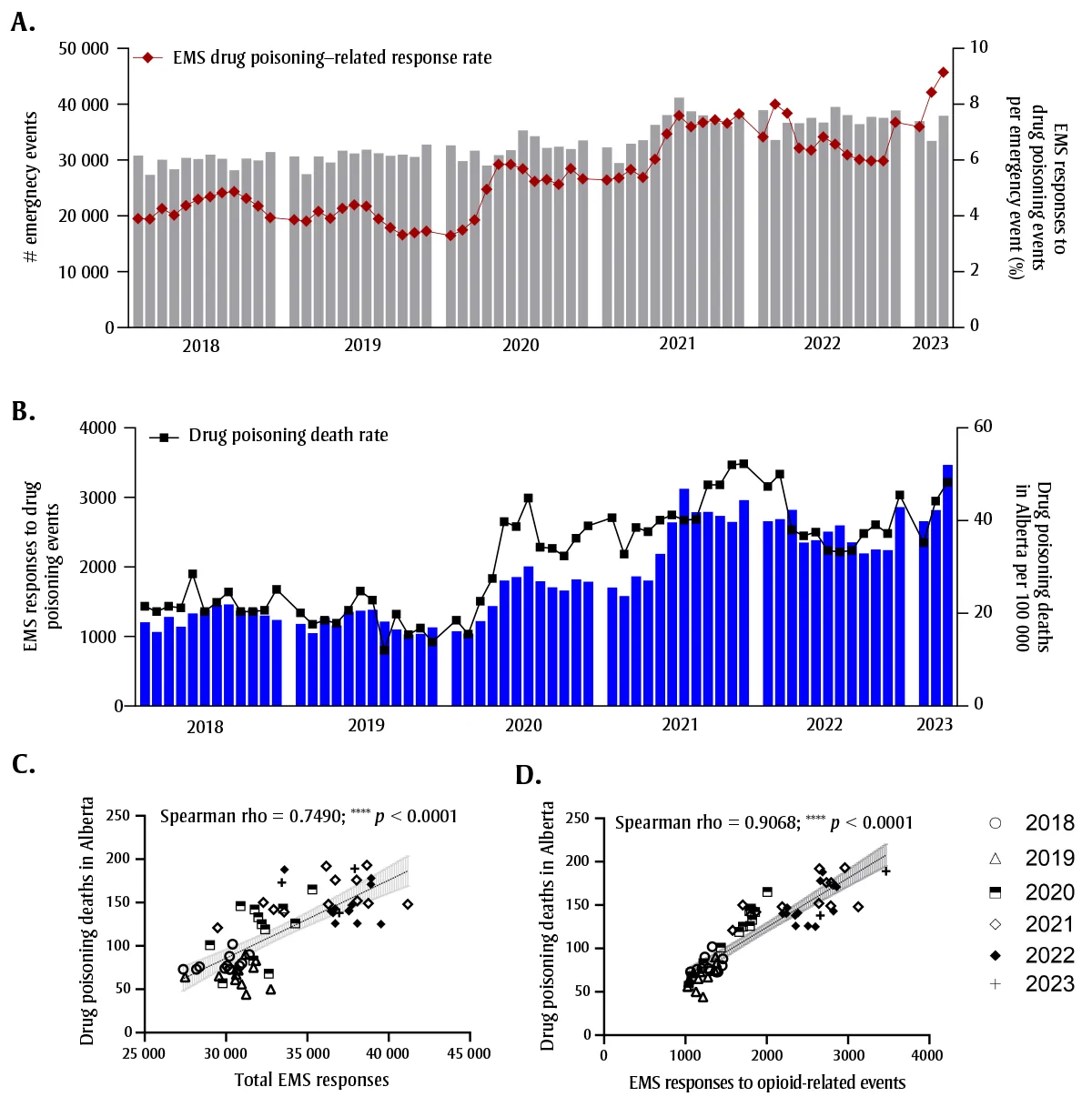
Associations between province-wide drug poisoning deaths and EMS dispatches in Alberta, Canada **Abbreviation:** EMS, emergency medical services. Notes: EMS dispatches positively correlate with drug deaths in Alberta from 2018 to 2023. **(A)** Monthly EMS dispatches for emergency events (grey bars; left y-axis) overlaid with EMS dispatches for drug poisoning events expressed as a percentage of total EMS emergency events (red diamonds; right y-axis) across Alberta from January 2018 to March 2023. **(B)** Monthly EMS dispatches for opioid-related events (blue bars; left y-axis) overlaid with the drug poisoning death rate in Alberta (deaths per 100 000; black squares; right y-axis) from January 2018 to March 2023. **(C–D)** Scatterplots of Alberta drug deaths as a function of province-wide **(C)** total EMS responses and **(D)** EMS responses for opioid-related events. Individual data points correspond to monthly data collected from January 2018 to March 2023, with symbols representing different years. Regression lines were fitted using a generalized linear model and error bars indicate 95% confidence intervals. Associations were assessed with Spearman’s correlation, with the rho (ρ) value and *p* value indicated above each scatterplot.

## Multiple linear regression analysis

Overall total visits and total visits where drugs were consumed at the Calgary SCS were both associated with decreases in drug deaths in the province (β = −.03, 95% CI: −0.032 to −0.019; β = −.03, 95% CI: −0.035 to −0.020, respectively). However, increases in drug poisoning events at Calgary SCS were associated with increases in broader drug death rates in the province (β = .73, 95% CI: 0.57–0.88). An association was also found between Alberta-wide EMS dispatches for opioid-related events and drug deaths (β = .06, 95% CI: 0.05–0.06), suggesting EMS dispatches for opioid-related events mirror drug deaths in the province (Figures 1 and 2). Total dispatches (including for non-opioid related events) were associated with marginal increases in drug deaths (β = 0.01, 95% CI: 0.007–0.011; β = .01, 95% CI: 0.010–0.016, respectively) (Table 3).

**Table 3:** Regression coefficients showing associations between Calgary SCS utilization and EMS dispatches on drug poisoning deaths in Alberta

**Measure**	**Category**	**β coefficient (95% CI)^a^**
Calgary SCS utilization	Total visits	−0.025 (−0.032, −0.019)
	Total visits with drug consumption	−0.028 (−0.035, −0.020)
	Total drug poisoning events	0.727 (0.574, 0.879)
	Poisoning rate	0.136 (0.125, 0.146)
EMS dispatches	Total	0.009 (0.007, 0.011)
	Opioid-related	0.057 (0.050, 0.064)
	Not opioid-related	0.013 (0.010, 0.016)

**Source:** 2018–2023 Alberta Health Services SCS monthly reports for Calgary SCS, and Alberta Health Services – EMS Research and Science.

**Abbreviations:** EMS, emergency medical services; SCS, supervised consumption site.

^a^ All analyses were adjusted for seasonality.

## Discussion

Our analysis suggests that both EMS and SCS drug poisoning event data may serve as potential proxies to understand drug deaths in Alberta. We found significant positive correlations between monthly SCS drug poisoning events and provincial drug poisoning deaths, monthly SCS drug poisoning events and Calgary drug poisoning deaths, and EMS dispatches for opioid-related events and provincial drug poisoning deaths. Interestingly, although not an initial objective of the study, we found that total SCS visits were significantly negatively correlated with provincial drug poisoning deaths. Findings from the multiple linear regression also suggests that increased SCS drug poisoning events are significantly associated with provincial drug poisoning deaths. Using these alternative forecasting methods can help service providers, people who use drugs and their families to respond to changes in the drug supply, potentially preventing deaths. This is particularly important in Alberta, where public drug death reporting is delayed for several months and remains inaccurate for many months after release. The results also highlight an inverse relationship between provincial drug deaths and both total client visits and Calgary SCS visits where drugs were consumed. This finding agrees with previous research linking SCS access to prevention of drug toxicity deaths.^22^

Unpublished data held by the Alberta government and Recovery Alberta, the health authority that operates SCSs, also suggest that regular clients of SCSs have approximately half the number of hospitalizations for substance use–related incidents, including drug poisoning, than people who use drugs but do not attend SCSs regularly.^32^ Hospitalizations of regular SCS clients show approximately half the duration of less-regular clients, indicating lower severity of medical distress. The data also reveal that supervised consumption clients in Alberta have two- to four-fold higher opioid agonist treatment (OAT) initiation than non-clients who use drugs.^32^

Our analysis illustrates a rise in drug deaths across Alberta from 2018 to 2023 that coincided with a decrease in SCS visits from 2020 to 2023. In Alberta at the onset of the pandemic (2020 Q2), SCS utilization fell sharply in all sites, which coincided with a steep rise in the frequency of drug poisoning events per client visit to the sites. The rate at which drug poisoning events occurred under supervised consumption followed similar trends across sites in Alberta, indicating that the provincial drug supply was experiencing relatively similar volatility through this period. Several short-lived outliers in drug poisoning frequency under supervised consumption (e.g. Calgary from Q3 to Q4, 2021; Grande Prairie Q3, 2023) may indicate geographically limited perturbations in the drug supply during those periods. These findings coincide with existing research that shows drug poisoning events increased in Canada during COVID due to a combination of increasing supply toxicity and changes to service access due to public health measures.^12^ We observed declining SCS utilization over the study period, which may be explained by the closure of SCSs across the province.^33^^-^^35^ Shifts to inhalation drug use and related unmet service needs may also inform SCS utilization.^36^^-^^39^ Given international evidence of effectiveness and our finding that SCS visits were significantly associated with decreased drug deaths, it is imperative that SCSs are funded to meet population needs as part of the response to increasingly volatile unregulated drug supplies.^40^

To our knowledge, this is the first study to use province-wide EMS and SCS data to understand trends in provincial drug deaths. It provides further validation for the Ottawa study that used municipal EMS data to predict drug deaths.^21^ Our findings also suggest that EMS dispatch data can be used to understand drug deaths despite changes to the drug supply. This supports findings from a descriptive study from the United States that concluded that monitoring drug poisoning-related EMS data can provide “real-time” insight into drug poisoning trends.^41^ Our finding that SCS visits are significantly associated with decreased drug poisoning deaths is consistent with previous research that showed decreased drug deaths in neighbourhoods where SCSs were implemented.^22^ Our findings contribute to growing knowledge on the association between SCSs and drug deaths, making use of SCS data that could specifically highlight changes in the drug supply.

## Strengths and limitations

Our focus on a single province where EMS data are reported consistently lends rigour to our research. This study uses routinely collected and accessible sources of data on SCSs and drug poisoning in Alberta, including provincial EMS dispatch data. A previous Canadian report attempted to estimate the number of EMS dispatches in response to opioid-related events; however, variance in how EMS data are reported can be a limitation.^42^^,^^43^ EMS records could be misclassified, as drug poisoning emergency events are recorded by paramedics but not toxicologically confirmed. Meanwhile, emergency events that are resolved on-site and do not involve transfer to a hospital, such as events responded to by outreach workers or fire departments, would not be captured in these data. Caution should be taken in applying EMS data from the study period to current events, because the relationship between drug poisoning fatalities and EMS dispatches may diverge as tranquilizers become more prevalent in unregulated drug supplies, including Alberta. To date, provinces including British Columbia, Ontario and Saskatchewan have issued public health warnings around increasing non-fatal poisonings related to tranquilizers, while Alberta has not.^44^^-^^46^

Most of our SCS analysis focused on data from one site in Calgary, because there were limited SCS data available from the other sites during the study period. In 2024, detailed monthly reports from the Calgary site were removed from public access. From then on, only quarterly data were available from provincial sites, and these lacked critical details captured in the earlier Calgary data, such as the number of consumption events. The number of site visits at Calgary SCS comprises 20% to 85% of the total site visits across Alberta during the study period; the large variation in these percentages can be attributed to few SCS being operational in 2018 and various site closures from 2020 onward. Although the SCS may reflect urban trends, we recognize that these data may not reflect trends in other urban and rural areas of the province, given that there are differences in local drug supply in Alberta.^47^ Residual confounding may also be a source of bias, considering that other variables associated with both EMS dispatches, SCS utilization, and drug deaths likely exist but were not available in the existing data. Given the ecological study design, we caution readers against equivalizing the current study risk estimates on individuals.

Future research should incorporate province-wide SCS data to provide a more comprehensive understanding of these relationships and validate our findings using data from other SCSs in Alberta or Canada. Researchers may consider assessing the correlation between EMS dispatches and drug deaths at a national level through the creation of a dependable case definition. Modelling differences based on gender, Indigeneity, and race would also be valuable, particularly given that key subpopulations are disproportionately affected by drug poisoning.

## Conclusion

Our study found that EMS dispatch data and SCS drug-related event data can be used to understand drug deaths on a provincial level. This is valuable learning for communities who wish to understand trends in drug deaths to respond effectively to the toxic drug crisis. However, this method is not a replacement for timely and transparent death data. Provincial and territorial governments must provide publicly available data on the unregulated toxic drug crisis to guide urgent responses to this ongoing public health emergency.

## Data Availability

None.
